# Exploring the Xylariaceae and its relatives

**DOI:** 10.1186/s40529-023-00389-6

**Published:** 2023-06-14

**Authors:** Nuttika Suwannasai, Ek Sangvichien, Cherdchai Phosri, Sirirath McCloskey, Niwana Wangsawat, Pisit Thamvithayakorn, Nutthaporn Ruchikachorn, Surang Thienhirun, Sureewan Mekkamol, Prakitsin Sihanonth, Margaret A. Whalley, Anthony J. S. Whalley

**Affiliations:** 1 Department of Microbiology, Faculty of Science, Srinakharinwirot University, 114 Sukhumvit 23, Wattana District, Bangkok, 10110 Thailand; 2 Department of Biology, Faculty of Science, Ramkhamhaeng University, Hua Mark Bangkapi, Bangkok, 10240 Thailand; 3 Department of Biology, Faculty of Science, Nakhon Phanom University, Nakhon Phanom, 48000 Thailand; 4 National Products Research Unit, Centre of Excellence for Innovation in Chemistry (PERCH-CIC), Department of Chemistry, Faculty of Science, Khon Kaen University, Khon Kaen, 40002 Thailand; 5 Food Research Unit, CPF Food Research and Development Center, 359 Moo 4 Wang Noi, Phra Nakhon Si Ayutthaya, 13170 Thailand; 6 The Institute for the Promotion of Teaching Science and Technology (IPST), 924 Sukhumvit Road, Phra Khanong Subdistrict, Klong Toei District, Bangkok, 10110 Thailand; 7 Department of Royal Forest, Forest Products Research Division, Bangkok, 10900 Thailand; 8 Plant Protection Program, Faculty of Agricultural Production, Maejo University, 63 Sansai-Phrao Road, Nongharn, Sansai District, Chiang Mai, 50290 Thailand; 9 Department of Microbiology, Faculty of Science, Chulalongkorn University, Bangkok, 10330 Thailand; 10 School of Pharmacy and Biomolecular Science, Liverpool John Moore University, Liverpool, L3 3AF UK; 11 National Centre for Genetic Engineering and Biotechnology, 113 Thailand Science Park, Phahonyothin Road, Khlong Luang, Bangkok, 12120 Pathumthani Thailand

**Keywords:** Xylariales, Xylariaceae, Systematics, Endophytes, Chemical profiles, Ecological aspects, Plant diseases, Host and habitat preferences

## Abstract

The Xylariaceae and its relatives rank as one of the best-known members of the Ascomycota. They are now well recognized for their diversity, global distribution, ecological activities and their outstanding novel metabolites with wide ranging bioactivity.

## Introduction

Professor Jack Rogers in his presidential address to the Mycological Society of America entitled ‘The Xylariaceae: systematics, biological and evolutionary aspects’ (Rogers 1979) triggered an on-going fascination of a previously underestimated family of the Ascomycota. The current authors are proud and honoured to be his followers. In his stimulating Benefactors’ Lecture to the British Mycological Society ‘Thoughts and Musings on tropical Xylariaceae’ he highlighted a number of aspects concerning current knowledge and missing information. (Rogers 2000). These included atypical morphological characteristics in certain accepted genera, their relationship to water conservation, their components of ecosystems, speciation and geographical distribution in the tropics. In relation to tropical Xylariaceae he agreed with Corner (1993) that future investigations of tropical fungi should be by resident tropical mycologists since much of importance is missed by short term collectors. Rogers also stressed the importance and value of holding workshops in the tropics where specialist mycologists could transfer their knowledge and experiences to willing students. The current appreciation of the family draws on many of the points he raised and herein we attempt to fill in some of the gaps and to answer several questions he raised.

### Systematic arrangement

Considerable changes to our understanding of the Xylariaceae and closely related families have occurred since the Publication of ‘A monograph of the world species of *Hypoxylon’* (Miller 1961) and the epic publications on *Xylaria* and relatives (Dennis 1956, 1961, 1970). Miller subdivided *Hypoxylon* into four sections *Hypoxylon*, *Papillata*, *Annulata* and Applanata with section *Papillata* subdivided into *Papillata* and *Primocinerea*, His designation of the species was based entirely on morphological features of their teleomorphs. The introduction of electron microscopy and use of anamorphic characteristics resulted in the recognition that many of the taxa belonged elsewhere. Many of the Applanata, when observed by scanning electron microscopy, were found to have ornamental spores of varying types (Rogers 1977). This resulted in these species being transferred to the genus *Camillea* Fr. (Læssøe et al. 1989) and further newly described species of *Camillea* have since been added (Rogers et al. 1991; Whalley et al. 1996, 1999). Major changes to sections *Papillata* and *Annulata* also followed with applanate species possessing dark, coloured ascospores with germ slits being accommodated in the genus *Biscogniauxia* Kuntz whilst many of the *Primocinerea* were transferred to *Nemania* S.F. Gray (Pouzar 1985a, b). *Jackrogersella* L. Wendt, Kuhnert, & M. Stadler was later erected for those species formerly included in *Hypoxylon* sect. *Annulata* Ju and Rogers (1996) and the *Annulohypoxylon* Y.-M. Ju, J.D. Rogers & H.-M. Hsieh to accommodate the species with papillate ostioles and lacking very conspicuous ostiolar disks (Wendt et al. 2018). The situation regarding *Xylaria* Hill ex Schrank and its allies is more complex; at present there is no monograph of the genus *Xylaria* but, it is likely to be composed of over 500 species (Rogers pers. comm.). In attempts to make their identification more manageable, the genus was arranged in subgroups based on size of the stromata (Rogers and Ju 2012) and for leaf and petiole inhabiting Xylarias three groups were recognized (1) the *X. filiformis* group; (2) the *X. phyllocharis* group and (3) the *X. heloidea* group based on stromatal shape and/or the conspicuousness of perithecial mounds on the stromatal surface (Ju and Hsieh 2023). The appreciation that presence or absence of chemical components was of taxonomic value (Whalley and Edwards 1995) initiated wide ranging studies on the chemicals present in the Xylariaceae in the discovery of numerous novel compounds as summarized by Marc Stadler and his colleagues (Stadler et al. 2004; Stadler and Hellwig 2005; Stadler and Fournier 2006). A major advancement was the introduction of high-throughput chemical profiling using HPLC profiles involving diode array detection and mass spectrometry resulting in a vast library of chemical components in a wide-ranging selection of taxa from the Xylariaceae (Stadler and Hellwig 2005). Early molecular phylogenetic investigations of the Xylariaceae were solely based using DNA loci such as the internal transcribed spacer region (ITS) of the nuclear ribosomal DNA (rDNA) (Sanchez-Ballesteros et al. 2000; Triebel et al. 2005; Pelaez et al. 2008). The ITS region is generally accepted as the standard barcode of fungi and has proved useful in the identification and separation of closely related species of *Hypoxylon* (Suwannasai et al. 2013b). However, a number of studies found the application of ITS data alone failed to resolve the taxa in the hypoxyloid clades as a result of its limitations as a phylogenetically informative locus. Hsieh et al. (2005) focussing on *Hypoxylon* and allies and later Hsieh et al. (2010) targeting the xylarioid clades discarded ITS finding a better resolution when based on the protein-coding genes alpha-actin (ACT) and beta-tubulin (TUB2). Tang et al. (2009) were the first to apply a multigene phylogeny derived from a combination of rRNA and protein the coding genes ITS, LSU, and TUB2. Tang et al. (2009) also used the section “6–7” of the second largest subunit of the RNA-polymerase II gene (RPB2) often used for barcoding and phylogenetic purposes within the fungal kingdom. Unfortunately, there were many gaps in their phylogeny and shortcomings in the selection of specimens or use of non-verifiable sources of sequence data (Wendt et al. 2018). Later Daranagama et al. (2014, 2015) provided additional rDNA and RPB2 sequence data which addressed a number of these omissions. An impressive multigene phylogeny which followed, included key representatives of the main lineages in the Xylariaceae, provided a clear vision of the Xylariaceae segregated into several major clades (Wendt et al. 2018).

**Fig. 1:**
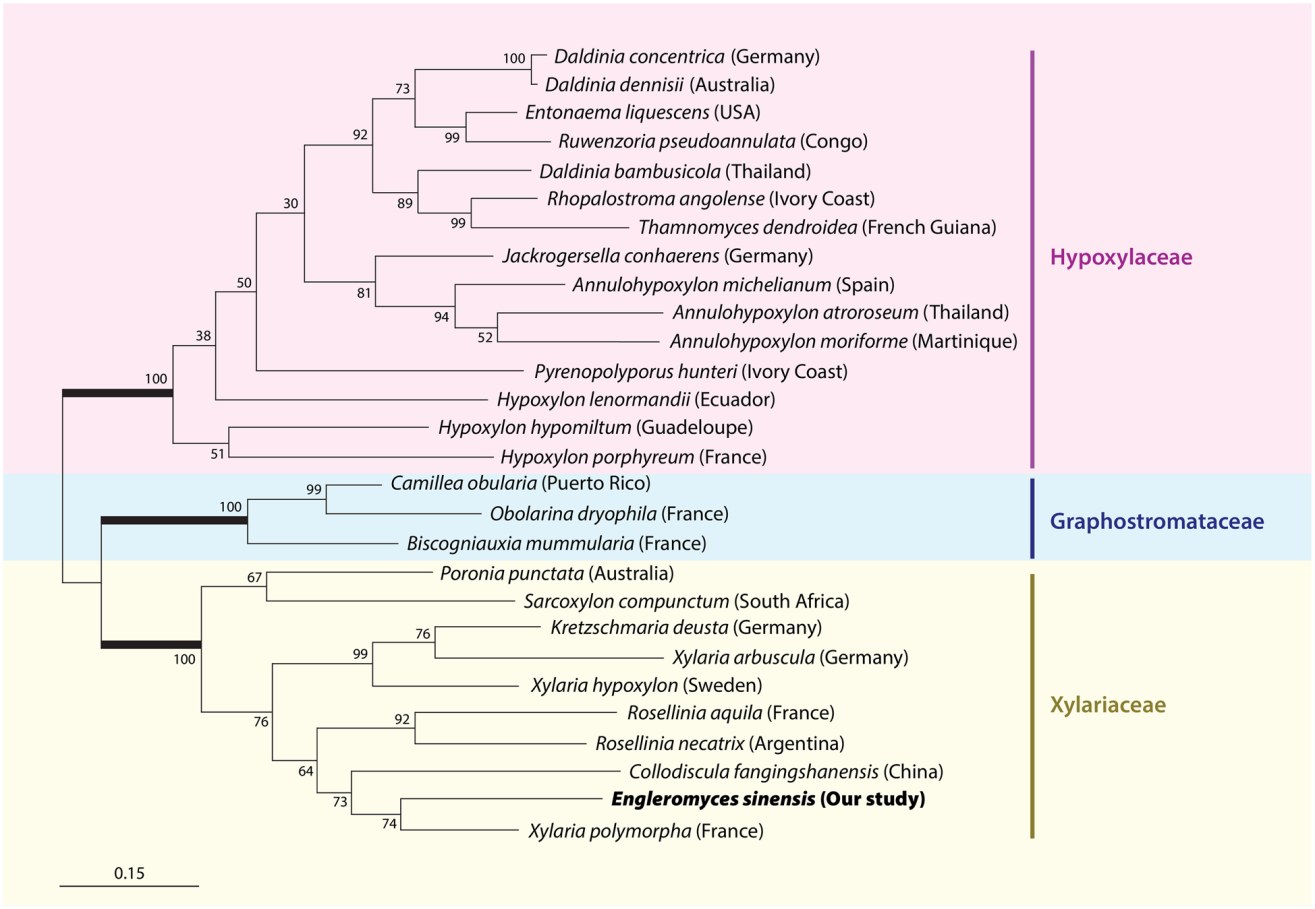
Phylogenetic tree based on the combined analysis of LSU (large subunit ribosomal ribonucleic acid) and *rpb2* (the second large subunit of RNA polymerase II) sequences based on maximum likelihood analysis. (Sangvichien et al. 2021, unpublished)

The currently accepted systematic arrangement of the Xylariales based on this multigene phylogeny consists of the families Diarypaceae, Graphostromataceae, Hypoxylaceae, Lopdadostomataceae and Xylariaceae s.str. Detailed explanation of these proposals together with chemical and morphological characteristics, which are in close agreement with the molecular findings, provides a more stable taxonomy of the Xylariales (Wendt et al. 2018). There is, however, the problem of acquiring modern or fresh collections of a substantial number of genera which echoes the admirable comments of Corner (1993) and Rogers (2000) of the need to have resident mycologists in tropical countries. It is, therefore, encouraging to report that the position of *Engleromyces* Henn. is now settled (Zhou et al. 2021) and their findings agree with our own. *Engleromyces sinensis* fits closely in the clade containing species of *Xylaria*, *Rosellinia*, *Kretzschmaria* Fr. and *Collodiscula* Hino & Katumoto and places *Engleromyces sinensis* with *Xylaria polymorpha* (Pers.) Grev., which is its closest relative at a bootstrap value of 74 followed closely by *C. fangjingshanensis* Q.R. Li., J.C. Kang & K.D. Hyde, *R. necatrix* (Berl. ex Prill.) and *R. aqulia* (Fr.) De Not. (Sangvichien et.al. 2021 unpublished. Figure 1). This confirms its rightful inclusion in the family Xylariaceae and reduces one of the uncertain genera referred to by Rogers. There is still more to do since *Anthostomella* Sacc. has been shown to be heterogeneous based on a comprehensive molecular phylogenetic study involving four independent markers and that species are polyphyletic across Xylariaceae (Daranagama et al. 2016a). There are also other genera, such as *Nemania*, *Rosellinia*, *Stilbohypoxylon* Henn., and *Xylaria* which may not be monophyletic (Hsieh et al. 2010; Stadler et al. 2013; Daranagama et al. 2015; Rogers and Ju 2017). Subsequently *Rosellinia* was split into *Rosellinia* s.str and *Dematophora* R. Hartig was resurrected for those taxa possessing anamorphs with synamata (Wittstein et al. 2020). Recognizing that there have been major changes in the systematics of the Xylariales Helaly et al. (2018) updated current thinking and accepted 26 families.

The increased application of secondary metabolite profiling together with multigene sequence data, collection of material from tropical countries and from unusual habitats has resulted in the discovery of many new xylariaceous taxa since ‘Thoughts and musings on the Xylariaceae’ (Rogers 2000). New species resulted from examination of ‘species complexes’ for example the *Hypoxylon fuscum* complex. Lambert et al. (2021) subsequently examined collections of *H. fuscum* (Pers.) Fr. from Iran and Europe comparing morphological, chemotaxonomic, and phylogenetic evidence and described the new species of *H. eurasiaticum* Pourmoghaddam, Krisal-Greihuber & Khodapand., *H. pseudofuscum* Pourmoghaddam, Khodap. In the UK *H. fuscum* usually grows on Corylus forming hemispherical to pulvinate stromata on bark or frequently effused-pulvinate stromata on decorticated wood. Collections made on Alnus or Betula deserve further examination since *H. pseudofuscum* appears to be strongly associated with Alnus, Quercus and Salix with *H. eurasiaticum* mainly associated with Quercus, thus, highlighting the importance of the host identity (Lambert et al. 2021). A good correlation had been found to occur between host type and ascospore dimensions in *H. fuscum* sl by Petrini et al. (1987) indicating a species complex and this has subsequently been supported by chemical and molecular data (Lambert et al. 2021).

### Environmental considerations

#### Host preference: specific or preferred

There are several species of Xylariaceae which can confidently be considered to exhibit host specificity although in many cases host preference is more appropriate. Examples of these include the bamboo inhabitants *Engleromyces goetzei* Henn., *E. sinensis* M.A. Whalley, Khalil, T.Z. Wei., Y.J. Yao & Whalley, *Xylaria badia,* and *Collodiscula* species. Petrini, in her scholastic monograph of *Rosellinia,* provided detailed records of species and hosts recognizing 21 species growing on bamboo (Petrini 2013). We elect, however, to use the term host preference since detailed examination of host relationships in the Xylariaceae identifies a broader host range than previously registered. In the Nordic countries host range for both *Jackrogersella multiformis* and *H. fuscum* greatly exceed their usually recognized host ranges (Granmo et al. 1989). In view of the recent chemical and molecular data on *H*. *fuscum*, it would be enlightening to examine any taxa which display a wide host range, or which grow on previously unrecorded hosts (Lambert et al. 2021).

There are *Hypoxylon* species which clearly have host preferences such as *H. fragiforme* (Pers.: Fr.) J.Kickx on Fagu*s*, *H. cercidicola* (Berk. & M.A. Curtis ex Peck) Y.-M. Ju & J.D. Rogers and *H. intermedium* (Schwein: Fr.) Y.-M. Ju & J.D. Rogers on Fraxinus and several others as detailed in the monograph (Ju and Rogers 1996). Other examples include *Xylaria longipes* Nitschke mainly found on Acer and* Jackrogersella cohaerens* (Pers.) L.Wendt, Kuhnert, & M. Stadler on Fagu*s* There are also examples where the species are seen to be faithful to their host tree species. *Dematophora buxi* (Fabre) C. lambert, K. Wittstein & M. Stadler has been reported as occurring on *Buxus sempervirens* and is distributed in Southern France and Great Britain (Petrini 2013). However, it also occurs on the evergreen box *B. colchinica* in the Ritsa Strict Nature Reserve located in the mountainous part of Abkhazia in the southern part of the Greater Caucasus range, Georgia (Whalley and Hammelev 1988). Recently, it has been recorded from Iran, growing on *B. sempervirens* and is a new record for Asia (Pouroghaddam et al. (2023).

It is also important to note that Rogers et al. (2002) considered seed and fruit-inhabiting species of *Xylaria* to be highly host-specific. Thus, *Xylaria carpophila* Fr. grows on fallen beech cupules, *X. oxyacanthae* Tul. on *Crataegus* seeds, *X*. *magnoliae* J.D. Rogers and *X. magnoliae* var. *microspora* J.D. Rogers, Y.-M. Ju & A.J.S. Whalley on *Magnolia* species, *X. liquidambar* J.D. Rogers, Y.-M. Ju & F. San Martin on *Liquidambar*, *X. jaliscoensis* San Martin, J.D. Rogers & Y.-M. Ju on fruits of Leguminosae (Rogers et al. 2002; Whalley 1996). Undoubtedly, knowledge of host specificity will change with the increasing number of studies on the Xylariaceae taking place.

### Habitat

#### Wood inhabitants and decomposition

Most family members are wood inhabitants and are reported as developing stromata on fresh fallen wood or sometimes even on branches still attached to their host tree. Chesters (1950) referred to these as primary invaders in the succession. In Fagus *H. fragiforme* is an early colonist and has been found to develop from pockets of decay within the wood where each pocket contained a genetically different individual (Chapela and Boddy 1988a). They were present within the wood as inconspicuous fungal propagules which then developed as ‘latent invaders’ once the high-water content of the wood was reduced (Chapela and Boddy 1988a, b; Whalley 1996). There are, however, some species occurring only on well-rotted and usually water-soaked decorticated wood such as *Nemania confluens* (Tode) Læssøe & Spooner ex Fr. West. and *N. uda* (Pers.) Gray. Interestingly both species are associated only with Quercus (Whalley 1996).

Various studies on wood decay by the Xylariaceae have shown that they can cause considerable weight loss and some species degrade lignin (Sutherland and Crawford 1981) whilst others exhibit strong production of cellulases (Wei et al. 1992). Few Xylariaceae colonize coniferous wood with species of *Rosellinia* being prevalent (Rogers 1979; Whalley 1985). Their preferential degradation of the syringylpropane units of lignin might explain their general absence from coniferous trees (Nilsson and Daniel 1989).

#### Seeds and fruits

Species occurring on seeds and fruits are mainly from the genus *Xylaria*. Rogers (2000) discussed their high host selectivity and provided alternative views on their lifestyle. The substrates could, as Whalley (1996) suggested, act as very specific baits in the litter layer but the fungus must be universally present in the locality of their hosts. They might, however, persist as endophytes on other hosts but to date no endophytic relationships have been proven. Rogers (2000), therefore, considered these fungi to be highly selective saprophytes or latent pathogens. Examination of more than 100 beech cupules collected from their host tree or those fallen onto tarmac or stone surfaces in areas where *Xylaria carpophila* occurred in nearby litter and soil never developed the *Xylaria* even after lengthy periods in damp chambers nor could they be isolated directly from the cupules. Visually uninfected cupules sampled from litter developed the *Xylaria* stromata in the damp chambers within 5–6 weeks ‘incubation’ (Hearn 2000). This could well be the case for other *Xylaria* species on seeds and fruits but in contrast Rogers et al. (2008) using a similar approach isolated cultures of *X. oxyacanthae* from seeds of *Crataegus monogyna* caught prior to touching the ground or taken from the canopy and, although in low percentages, proved that the fungus infects the seeds while on the tree, probably by infecting the flowers. Later, Ju et al. (2018) stated that infection of the seeds appears to be through the flowers and that this is also the case for *X. magnoliae*, *X*. *liquidambar* and *X. carpophila* (Ju et al. 2018). There are, therefore, still unanswered questions and until molecular techniques are applied to follow propagules, and for infections to be traced, the jury remains out.

#### Leaves

There are species of *Xylaria* which invade living leaves and petioles often fruiting on living material. Ju et al. (2018) studied forty-five foliicolous and caulicolous taxa of *Xylaria* and described nine new species and three unnamed species on leaves. These were classified into three groups on the basis of their stromatal shape and on the level of the conspicuousness of their perithecial mounds: the *X. filiformis* group, the *X. phyllocharis* group and the *X. heloidea* group. Three new species of *Xylaria* on fallen leaves in Hainan Tropical Rainforest National Park have been recently described based on morphological and molecular evidence with each taxon occurring on a specific host by Pan et al. (2022). The three taxa described, *X. polysoricola* H.X. Ma & X.Y. Pan, *X. lindericola* H.X. Ma & X.Y. Pan and *X. hedyosmicola* H.X. Ma & X.Y. Pan are named after their host plants and were considered to be host specific. Thus, host specificity may prove to be an important feature of leaf and petiole inhabiting *Xylaria* species. This is also supported by the publication of three host specific *Xylaria* species from Puerto Rico with *X. meliacearum* Læssøe on petioles of Meliaceae, *X. guareae* Læssøe & Lodge on branches of Meliaceae and *X. axifera* Mont. restricted to petioles of plants in the Araliaceae (Læssøe and Lodge 1994). The authors also noted that *X. axifera*, although it is restricted to the petioles, apparently does not invade its host until after the leaves have fallen. This is perhaps a similar situation to those *Xylaria* species occurring on fallen fruits and seeds where there is no clear-cut evidence on their mode of infection. Is infection taking place in the leaves whilst still on the tree or does infection occur in the litter with the host material acting as a bait?

Pan et al. (2022) also pointed out that ‘Especially, the study of *Xylaria* species growing on fallen leaves or petioles is far behind those taxa associated with other substrates’.

#### Ant and termite nests

There are many *Xylaria* species associated with ant and termite nests and usually the stromata only appear once the nest has been abandoned. The relationship between the *Xylaria* and the insect is still not known but it is postulated that the insects cultivate the fungus for food (Rogers 2000). He also made it clear that there are undescribed species and that these need to be studied (Rogers 2000). Our recent studies in Thailand recognized 12 new taxa of *Xylaria* associated with termite nests from Northeast Thailand based on morphological and cultural characteristic and their ITS α-actin and β-tubulin sequences (Wangsawat et al. 2021a). This finding emphasises the importance of resident mycologists in the tropics to investigate and to conduct regular surveys in specified habitats.

#### Dung

Xylariaceous fungi exhibiting a special relationship with dung are members of the genera *Hypocopra* (Fr.) J.Kickx, *Podosordaria* Ellis & Holw., *Poronia* Willd. and *Wawelia* Namys. They are united by ascospores possessing dark walls, sticky sheaths, and multinucleate condition resembling those of their sordariaceous relatives (Rogers 2000). *Wawelia* is different and all five known species from Europe occur on mainly leporid dung associated with dry habitats (Webster et al. 1999). It has been suggested that this apparent rarity of *Wawelia* may be explained by its xerophilic nature and for its lack of records from field samples (Lundquist 1992). Usually, it is only recorded after incubation in damp chambers in the laboratory (Webster et al. 1999). Sadly, but perhaps, because of the short-lived appearance of their fruit bodies in the field, the dung Xylariaceae have been neglected, especially in the tropics. In their account of *Hypocopra* Krug and Cain (2011) reported on 14 new species of *Hypocopra* from 9 different animal dung samples recorded from a total of 12 different countries, including tropical Mexico and Kenya. *Stromatoneurospora phoenix* (Kunze) S C. Jong & E.E. Davis has now been shown to have affinities with the coprophilous Xylariaceae, based on morphological, molecular, and chemical data (Becker et al. 2020). This confirms the earlier suspicions of Rogers (2018) that *S. phoenix,* although an inhabitant of burnt grasses, is a relative of the coprophilous Xylariaceae and Becker et al. (2020) placed *S. phoenix* close to *Poronia* in their phylogeny. It did not, however, produce the punctaporonin metabolites present in *Poronia* (Anderson et al. 1988; Becker et al. 2020).

Rogers et al. (1998) described *Podosordaria elephanti* J.D. Rogers & Y.-M. Ju. on elephant dung from Chachoengsao Province in Thailand but as far as we are aware it has subsequently only been reported from India together with *Poronia pileiformis* (Berk.) Fr. on elephant dung (Deepa Latha and Manimohan 2012). Studies on coprophilous fungi in Thailand based on samples from 13 different animal species collected from 14 localities were undertaken using damp chamber incubation or soil plate isolation. A total of 68 isolates were obtained belonging to 12 genera and 15 species of coprophilous ascomycetes (Jeamjitt et al. 2007). Interestingly, *P. elephanti* was not recorded and *Podosordaria leporina* (Ellis & Everh.) Dennis was the only member of the Xylariaceae reported. Richardson (2021) stressed the importance of frequent sampling and recording at the selected sites and noted that some fungi preferred different types of animal dung such as horse, sheep, rabbit, or grouse.

### Adaptation to dry environments and strategies for water conservation

**Fig. 2:**
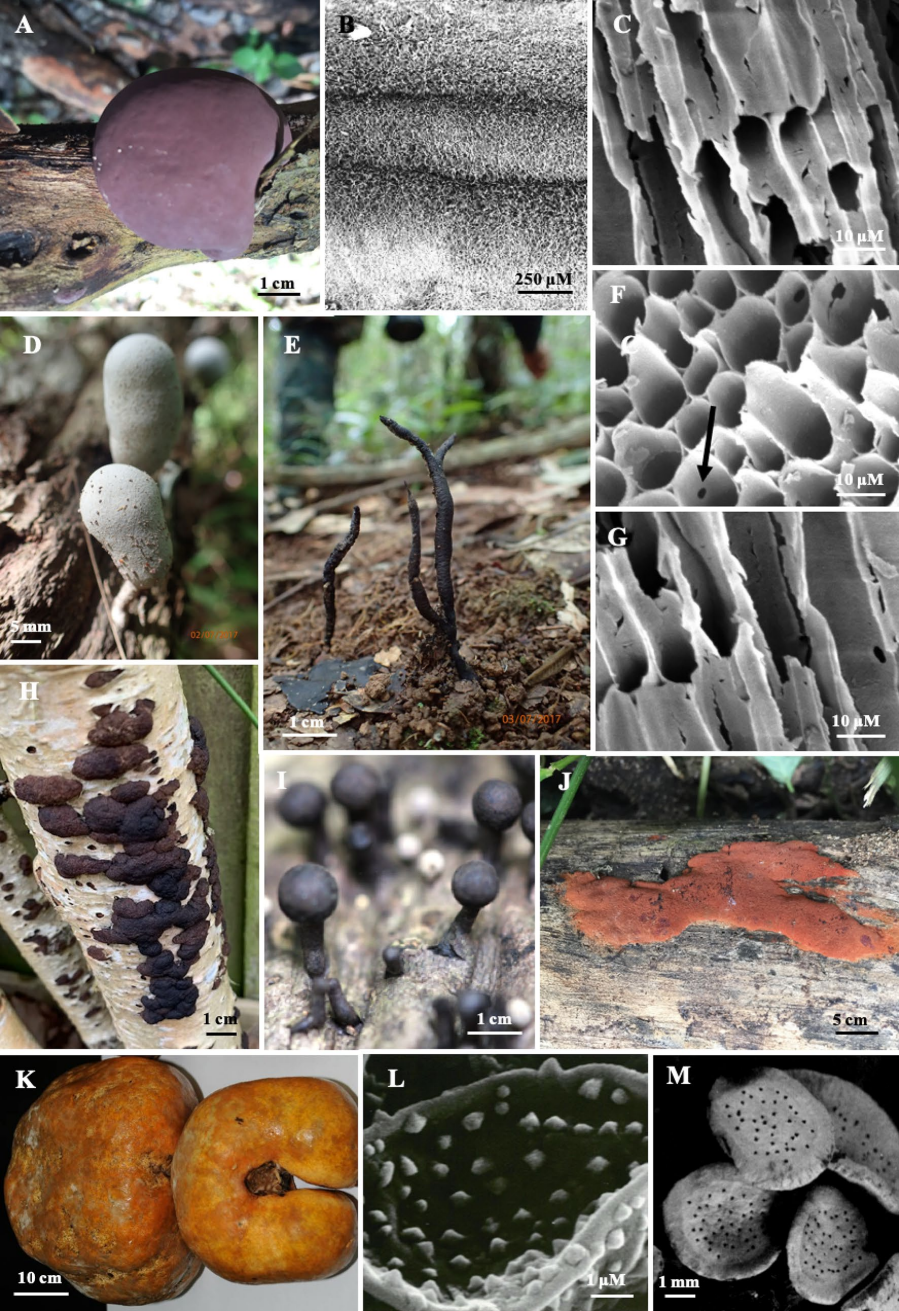
Images of Xylariaceae and relatives. **A**. Stroma of *Daldinia eschscholtzii*. **B**. Hyphal zonation in *Daldinia concentrica* (SEM). **C**, **F**, **G**, Hyphal elements from the three zones highlighting change in hyphal orientation in *D. concentrica*. D. *Xylaria fockei* (Miq.)Cooke Stromata. E. *X. margaretae* Wangsawat, N, Y.-M. Ju, Phosri, C, Whalley, A.J.S. and Suwannasai, N. stromata. H. *J. multiforme* stromata. I. *R. lekae.* stromata. J. *Hypoxylon haematostroma* Mont. K. *E. sianensis* stromata. L. *C. selangorensis*. M. *P. puncata*. Stromata

The recognition that members of the Xylariaceae are often associated with dry habitats was highlighted by (Rogers 1979; Whalley 1985, 1996). Rogers, in his Presidential address to the Mycological Society of America (Rogers 1979), hypothesized that various xylariaceous lifestyles developed on periodically dry sites and he summarized the different types of adaptations to the relationship of perithecial stromata to substrate. These included stromata embedded in decayed wood, stromata superficial but borne in a subiculum, stromata embedded in dung, stromata erumpent from bark via a dehiscent outer stromatal layer, the presence of gelatinous material or possessing massive stromata (Rogers 2000). The genus *Daldinia* Ces. & De Not. is characterized by concentric zonation and Ju et al. (1997) emphasized the importance of the zones and its xerophilic habitat indicating that the concentric zones in *Daldinia* stromata are, in their opinion, the key to its xerophilic habitat. They also stated that when the zones are initially formed, they tend to be dense and more or less gelatinous. As stromata age the zones lose their density and collapse or at least become riddled with lacunae’. Ju et al (1997) showed that these zones were not a result of aborted perithecia which agrees with Stadler et al. (2014) and is not a result of aborted perithecia as suggested by Bayliss-Elliot (1917). Light and scanning electron microscopy proved that zonation is, however, a result of the regular alternation of the zones caused by changes in orientation of hyphal growth (Khalil et al. 2015; Fig. 2). This would, in our opinion, slow down the movement of water from the stromal base still attached to the host and the regular finding of *Daldinia* species on large fallen trunks or on recently dead or dying trees in exposed or dry situations and is consistent with their xerophilic nature. *Daldinia concentrica* (Bolton) Ces. & De Not. on fallen Fraxinus, its usual host in the UK, has been observed to develop over several years to maturity in water stressed environmental conditions. In Europe *D. caldariorum* Henn*.* is only found on burnt and weathered gorse, *Ulex europaeus*, which is another water stressed environment (Khalil et al. 2015). In Thailand and Malaysia, it is usual to find *D. eschscholtzii* (Ehrenb.) Rehm growing on a log pile or on fallen trunks or large branches in clearings in the forest where they are exposed to the sun and therefore experience periodic drying (Whalley 1996). *Daldinia* species found in Papua New Guinea were restricted to open and extremely dry sun-exposed sites (Van der Gucht and Whalley 1996). *Entonaema* A. Möller is now recognized to consist of hypoxyloid and xylarioid species with the xylarioid forms being transferred to *Xylaria* (Stadler et al. 2008). It is often present in similar situations to *Daldinia,* occurring on large logs in dry habitats, and is closely related to *Daldinia* (Rogers 2018; Stadler et al. 2004).

The stromata are filled with liquid when fresh but become hollow when dry. Ju et al. (1997) argued that the water presumably fills an expanding cavity with gel lined walls and that it seems possible that *Entonaema* has dispensed with rings altogether, using turgor pressure generated by gels to both expand the stroma and store water. Both *Daldinia* and *Entonaema* exhibit an astute water regulation which enables them to develop to maturity.

Edwards et al (2003) summarized many reports about canker diseases caused by *Biscogniauxia* and *Camillea* in stressed trees growing in dry spots in a forest environment and is discussed herein under plant diseases. Furthermore, *Wawelia* species are associated with leporid dung and, as discussed under dung inhabiting Xylariaceae, *Wawelia* species are xerophytic and rarely found in the field being recorded in the laboratory following damp chamber incubation (Webster et al. 1999).

The significance of dry environments as a habitat for xylariaceous fungi in relation to the Xylariaceae is well shown by several surveys in Kuala Selangor Nature Park in Malaysia. The park contains a wide variety of habitats including mangrove forest, secondary forest, estuary, mud flats and brackish water lake system. Since the first survey in 1993 there has been a continuing change in the abundance of tree species as the water table has become lower. In the secondary forest where the surveys were carried out there has been a noticeable increase of species of strangler figs, *Ficus macrocarpa* L. becoming superseded by *F. gibbosa* Blume which is hardier and more aggressive (Whalley et al. 2002; Whalley and Whalley 2007). Over the 4 visits to the park (1993, 1995, 1997 and 2000) 35 taxa representing 8 genera of Xylariaceae were recorded. In the Park, certain taxa were associated with specific hosts or were only found in particular situations. *Daldinia eschscholtzii* only occurred on large fallen logs and our observations in Kuala Selangor Nature Park and at other sites in Malaysia and Thailand indicate that *Daldinia* occurs most frequently in open parts of the forest, in tree fall areas, by paths or in log piles – all drier sites within the forest ecosystem (Thienhirun 1997; Whalley et al. 1999; Thienhirun and Whalley 2004). The Park is also the type locality for *Camillea selangorensis* M.A. Whalley, A.J.S. Whalley & E.B.G. Jones which was collected in 1993. During the 1997 survey eight collections of *C. selangorensis* were obtained, all from freshly fallen branches of *F. macrocarpa* lying on the forest floor. Similar findings were obtained in the 2000 survey. Furthermore, as the secondary forest continued to become drier increased collections of *Hypoxylon*, *Biscogniauxia* and *Nemania* were made (Whalley et al. 2002).

Representatives of twelve genera of Xylariaceae have been recorded from mangroves in Malaysia and Thailand (Whalley et al. 1994; Chareprasert et al. 2012) and *Halorosellinia oceanica* (Schatz) Whalley, E.B.G. Jones, K.D. Hyde & Læssøe from Florida is now recognized as a common and widespread mangrove species (Whalley et al. 2000). A series of publications by E.B.G. Jones and K.D. Hyde have over the last 25 years provided new insight into marine and mangrove fungi. These include several new genera and species of the Xylariaceae (Hyde 1990, 2007; Whalley et al. 1994; Jones 2007; Jones and Kuthubutheen 1990; Lu and Hyde 2000). Species of *Anthostomella* Sacc. and *Nemania* are widely distributed mangrove fungi and a further eleven xylariaceous genera have been recorded (Chareprasert et al. 2012). Devadatha et al. (2021) reviewed the many publications regarding their taxonomy, occurrence, and geographical distribution.

### Ecological situation and lifestyle strategies

#### Xylariaceae and plant disease

Generally, the Xylariaceae are not strongly pathogenic but are basically facultative parasites being opportunistic and weakly pathogenic (Whalley 1996; Edwards et al. 2003; Rogers 2000, 2020). Diseases can be separated into needle blights, root infections and canker diseases.

Needle blights are linked with species of *Rosellinia* and young conifer trees growing in forest nursery beds. They can locally result in serious damage to the plants especially in overcrowded conditions where there is prolonged high humidity (Francis 1986; Ten Hoopen and Krauss 2006). Francis (1986) concluded that many of the reported outbreaks were caused by *R. minor* (Hohm.) S.M. Francis especially in crowded, humid conditions and not *R. herpotrichoides* Hepting & R.W. Davidson as was frequently cited as the culprit. *Rosellinia herpotrichoides* is noted to occur on *Tsuga* branches in more open situations rather than in overcrowded and humid environments and is restricted to the USA (Petrini 2013).

Root rot infections are caused by a very diverse range of Xylariaceae with *Dematophora* and *Kretzschmaria* species being the most widespread and economically important. *Kretzschmaria clavus* (Sacc.) Fr. causes a serious root decay in macadamia (*Macadamia intergrifolia* Maiden, & Betche) in Hawaii (Ko et al. 1977) and Taiwan (Ann and Ko 1988). *Kretzschmaria clavus* is widespread in the tropics and subtropics (Whalley 1993, 1996) and occurs on a wide variety of forest trees which led to the speculation that these provided an infection source for the macadamia (Ko et al. 1986). Another species, *K. zonata* (Lév.) P.M.D. Martin has long been recognized as causing a serious root rot in rubber trees (Vargese 1971) and has since been reported to be an important problem in South Indian rubber plantations although it could be controlled by application of fungicides and petroleum wound dressings (Idicula et al. 1990). In Britain *K. deusta* (Hoffm: Fr.) P.M.D. Martin causes rot at the bases of a variety of tree trunks (Burdekin 1977) and can become locally of economic significance. In the Sara mountains of Yugoslavia an infection rate of up to 42% in beech trees has been observed in some localities (Prljincevic 1982) and in Czechoslovakian forests 11–20% of beech trees were infected by this fungus (Cerný 1970; 1975). Species of *Dematophora* are a major cause of white root rots or brown rots in numerous plant species (Sivanesan and Holliday 1972a, b,). *Dematophora necatrix* R. Hartig is reported as a plurivorous pathogen with world-wide distribution (Sivanesan and Holliday 1972b).

Canker diseases of trees are usually linked to drought conditions or to damage caused by insects e.g., *Entonaema mammata* (Wahlenberg) J.D. Rogers & Y.M. Ju or previous infections by wilt causing fungi. In quaking aspen, the gall forming insect *Saperda inornata* provides infection wounds for *E. mammata* (Anderson et al. 1979) or canker by *Biscogniauxia nothofagi* Whalley, Læssøe & Kile on *Nothofagus cunninghamii* previously infected by *Chalara australis* Walker & Kile (Whalley et al. 1990). Many of the fungi involved in these canker diseases are former members of the Xylariaceae belonging to the genera *Biscogniauxia* O. Kuntze and *Camillea* Fr. but now placed in the Graphostromataceae (Wendt et al. 2018). Canker diseases caused by members of these two genera have been reviewed with indications of strong associations with water stressed hosts (Edwards et al. 2003).

*Entoleuca mammata* (Wahlenberg) J.D. Rogers & Y.M. Ju is, however, an important cause of canker in quaking aspen (*Populus tremuloides* Michx) responsible for a major loss of young trees in the forests of the Lake States of the USA (Manion and Griffin 1986). Marty (1972) attempted to determine the cost of *Hypoxylon* canker in the region and indicated at the time of harvesting the loss amounted to US$4.4 million per year. Although the Xylariaceae are not normally regarded as the cause of plant diseases there are a number of species which result in considerable economic loss and from time to time there are reports, often local, of significant damage.

In many of the diseases there is a clear relationship between the host and water stress. Several species of *Biscogniauxia* and *Camillea* cause cankers in water stressed trees. In cork oak and *B. mediterranea* (De Not.) Kuntze in Southern Europe (Vannini and Scarascia Mugnozza 1991), and *B. nummularia* (Bull: Fr.) Kuntze) with beech in Sicily (Granata et al. 1996). In the southern states of North America *B. atropunctata* (Schwein.) Pouzar causes a serious drought related disease on oaks (Thompson 1963; Bassett and Fenn 1984). Also, in the USA, *Camillea punctulata* (Berk. & Rav.) Læssøe, J.D. Rogers and Whalley infects oak in south eastern U.S.A. where it is linked with trees which are water stressed following prior infection with the oak wilt fungus *Ceratocystis fagacearum* (Bretz) J. Hunt (Davis 1964) and *C. tinctor* (Berk.) Læssøe, J.D. Rogers and Whalley is associated with canker of American sycamore (McAlpine1961) and plane (Hepting 1971). Perhaps the strangest association, however, must be that observed between *Hypoxylon rubiginosum* (Pers.) Fr. and catalpa which was reported from the campus of the University of Georgia USA (Weidell 1942). Local fishermen obtain the catalpa worm (*Ceraomia catalpae*) for bait by beating the trunks of the trees with clubs to dislodge them, thus causing localized injury leading to the development of cancer caused by *Hypoxylon* (Weidell 1942).

### Endophytes

Over the past several decades there has been a plethora of publications on endophytes with members of the Xylariaceae prominently reported (Petrini and Petrini 1985). A study following the development of endophytes in leaves of *Tectona grandis* L. (teak) in Chang Mai Province at the beginning of the rainy season endophytic Xylariaceae proved to be the most frequent (Mekkamol et al. 1997; Mekkamol 1998). *Daldinia eschscholtzii*, *N. bipapillata* (Berk. & M.A. Curtis) Pouzar, *H. haematostroma* (Mont) Fr.. and *X. cubensis* (Mont.) Fr. were frequent isolates with *Daldinia* being detected early in the rainy season and *Xylaria* species occurred later. The overwhelming number of publications on endophytes makes it impossible to do them justice here. This major interest in endophytes was certainly stimulated by the discovery of many novel compounds and the promising leads in new drug discovery (Helaly et al. 2018). There was also the earlier discovery that the anti-cancer drug, taxol, was produced by the endophyte *Taxomyces andreanae*.Srobel, A. Stierle, D. Stierle, & W.H. Hess from *Taxus brevifolia* Nutt., Pacific yew (Stierle et al. 1993). This was later shown to belong to the basidiomycete genus *Ceriporiosis,* not to a hyphomycetous genus, and was re-assigned as *C. andreanae* (Strobel et al. 1996) T. Cheng and M. Stadler (Cheng et al. 2022). The number of reports on metabolites from endophytic Xylariaceae and relatives, coupled with their recognition as a major source of novel chemicals with bioactive properties, has undoubtedly been the driving force stimulating these investigations (Stadler and Hellwig 2005; Becker and Stadler 2021; Wongkanoun et al 2020). Endophytic Xylariaceae are undoubtedly widespread worldwide and have been isolated from a very diverse host range and their track record as a source of many novel and bioactive compounds will ensure many more studies will follow. Becker and Stadler (2021) noted that many of the recent discoveries of novel metabolites originated from endophytic isolates of *Xylaria*. It is noteworthy that although endophytic Xylariaceae and their relatives appear almost ubiquitous the situation of the host plants sampled is of primary importance. A study of endophytes on *Cassia fistula* L. (golden shower) in Thailand involved sampling in 3 geographical sites to allow comparisons between their endophytic assemblages and to evaluate these data in relation to differences in plant diversity, density and the local environment. Overall, members’ species of *Xylaria* and *Daldinia* were most frequently isolated but representatives of *Nemania* and *Hypoxylon* were also recorded (Ruchikachorn 2005). The Kanchanaburi site, being closest to a natural forest situation, provided the highest number of isolates whereas the Bangkok site, characterized by isolated individuals, yielded least (Ruchikachorn 2005). Surveys of mangrove forest tress growing in different locations (Chanthaburi Province, Prachuap Khiri Khan Province and Rayong Province in Thailand) revealed that the dominant endophytes varied by host type. *Phyllosticta* was the most frequently isolated endophyte, but several different *Xylaria* species were obtained from several tree species. Although the percentage frequency of *Xylaria* was low, *D. eschscholtzii* was, however, a regular isolate from Ranong mangrove achieving 7.33% on *Xylocarpus granatum* Koen. and 6.67% on *Rhizophora apiculata* Bl. of all isolates (Chareprasert et al. 2010). Screening of crude extracts for antimicrobial and toxicity against a selection of cancer cell lines suggested that investigation of the metabolites from some isolates would be worthwhile (Chareprasert et al. 2010; Sujarivorakol et al. 2011).

### Geographical distribution

Assessing global distribution of fungi is fraught with problems as fungi are ephemeral and often their records are based on the collector being in the right place at the right time (Watling 1978). The importance of season in relation to rainfall in the tropics was stressed by Corner (1993). Whilst this is certainly true of the fleshy fungi of the Basidiomycota many of the Ascomycota are more resilient and exhibit extended ‘fruiting’ periods and are therefore more persistent in nature. Many members of the Xylariaceae have a carbonaceous stroma, are more robust or have developed strategies for inhabiting dry environments and this, coupled with a considerable increased interest in the family and its allies increases the likelihood that xylariaceous fungi are recorded. It does, however, need to be emphasized that their distribution records are mainly based on the presence of their teleomorphs and as they appear to be almost ubiquitous as endophytes this is an important consideration. Caution is also required when comparing distribution between regions and there is a need to consider how many different habitats were surveyed and how frequently; collector bias is often a problem (Van der Gucht and Whalley 1996). As stressed by both Corner (1993) and Rogers (2000) the importance of resident mycologists well acquainted with the Xylariaceae is an important consideration. This is well supported by the impressive collecting and publications by K.D. Hyde and his co-workers in S.E. Asia and China over a 20 year period. This has greatly increased our understanding of the Xylariaceae and related families (Daranagama et al. 2016b).

Despite these current limitations several distribution patterns can now be recognized with some confidence (Van der Gucht and Whalley 1996; Suwannasai et al. 2013a). These have been supported by monographs of many genera, increased surveying and application of molecular techniques to clarify species complexes (e.g*., H. fuscum*). *Xylaria* is the only major genus awaiting a World monograph. In certain genera distribution is tropical or subtropical whilst others occur in temperate countries. There are also those found mainly in either the North or South hemispheres. *Camillea* has for many years been considered generally restricted to the Amazon region. Now that there is a modern monograph, several species placed in *Hypoxylon* (Miller 1961) were transferred to *Camillea* and they occur in Mexico and the southern states of the U.S.A. (Læssøe et al. 1989). The publication of two new species, *C. selangorensis* M.A. Whalley, A.J.S. Whalley & E.B. G. Jones (Whalley et al. 1996) and *C. malayasianensis* M.A. Whalley (Whalley and Whalley 2007) has shed new light on distribution outside of the Americas. These two new *Camillea* species originated from the Kuala Selangor Nature Park (Malaysia) in secondary forest where the mangrove is in decline (Whalley et al. 2002). *Camillea selangorensis* has since been recorded from Thailand in secondary forest in Phuket also bordering mangroves (Whalley et al. 1999). *Camillea tinctor* is the one species which has a widespread distribution (Læssøe et al. 1989; Whalley et al. 1999). Miller reported on a collection of *C. tinctor* from Singapore as the applanate *Hypoxylon tinctor* but was doubtful about its origin (Miller 1961). However, more recent collections from Papua New Guinea (Van der Gucht 1995), Malaysia and Thailand (Thienhirun 1997; Whalley et al. 1999) confirm that it is truly pantropical and probably common. It was recorded from eight separate localities in Thailand, six in Malaysia and ten collections from Papua New Guinea (Whalley et al. 1999; Van der Gucht and Whalley 1996) and from Taiwan (Ju 2000).

There are also those species which follow their host plant species. *Annulohypoxylon bovei* (Speg.) Y.M. Ju, J.D. Rogers & H.M. Hsieh was originally described from Argentina, but is also known from Australia, Indonesia, and New Zealand, probably associated with Nothofagus species (Ju and Rogers 1996). *Xylaria castorea* Berk. is known from South America and New Zealand and ‘it seems probable that both of these species have been distributed along with Nothofagus in part by repositioning of land masses’ (Rogers 2000). Surprisingly, *A. bovei* has been reported from the Hawaiian Islands on *Eucalyptus,* not the usual *Nothofagus* (Rogers and Ju 2012).

*Dematophora buxi* Fabre is another species faithful to its host being recorded from France, UK and Georgia in the former USSR (Petrini 2013; Whalley and Hammelev 1988). Examination of mature box trees in other countries may prove to be worthwhile. Host and locality are also important. When *Engleromyces goetzei* Henn. was described from East Africa in 1890 it immediately became recognized as one of the largest and most spectacular pyrenomycetes known*.* It remained the only species in the genus until *E. sinensis* M.A. Whalley, A. Khalil, T.Z.Wei, Y.J. Yao and A.J.S. Whalley was added in 2010 (Whalley et al. 2010). Earlier records of *E. goetzei* from China proved to be *E. sinensis* although interestingly both species occur on mountain bamboo at altitudes of over 2000 m above sea level*.*

*Hypoxylon fragiforme* occurs on Fagus in temperate regions but the very similar but smaller spored *H. howeianum* Peck has a cosmopolitan distribution on a variety of hosts (Ju and Rogers 1996). *Jackrogersella cohaerens* is also common on Fagus in temperate regions whilst the smaller spored *J. cohaerens* var. *microsporum* has a wide geographic and host range (Ju and Rogers 1996; Wendt et al. 2018). Rogers speculated that the presence of species with larger ascospores in the cooler regions might be associated with food reserves of the larger ascospores and longer periods of dormancy in cooler environments (Rogers 2000).

We are reluctant to list species which might be endemic and although there are many which at present are only known from their type locality a number, which in the past were considered to be endemic, or at least restricted, might have been included, but have subsequently been found in quite distant localities. Thus, *H. vandervekenii* first described from Papua New Guinea (Van der Gucht et al. 1997) has now been collected in the Hawaiian Islands (Rogers and Ju 2012) and *B*. *anceps* which was originally reported from Italy is widespread in France, occurs in the British Isles, Honduras (Rogers et al. 1996) and Mexico (Ju et al. 1998). We are fully in agreement with Trierveiler-Pereira et al. (2009) who stated that ‘Although *Xylaria* is considered one of the best-known genera in the family nearly 65 new species of *Xylaria* have been described during the past 20 years, 25 of which have been described since the year 2000’ and our data shows the importance of continuous studies on the genus, especially in the tropics, where *Xylaria* diversity is very high. Over a similar period, impressive details are given on new genera of Xylariaceae (Wendt et al. 2018). These include *Brunneiperidium* Daranag., Camporesi and K.D. Hyde, *Coniolariella* Dania Garcia et al., *Emarcea* Duong, Jeewon and K.D. Hyde, *Halorosellinia* Whalley, E.B.G. Jones, K.D. Hyde and T. Læssøe*, Lunatiannulus* Darang*.,* Camporesi and K.D. Hyde, *Anthocanalis* Darang., Camporesi and K.D. Hyde, *Alloanthostomella* Durang., Camporesi & K.D. Hyde, *Appendixia* B.S. Lu and K.D. Hyde, *Camporesia* W.J. Lu and K.D. Hyde, *Cannonia*, Joanne E. Taylor and K.D. Hyde. It should be noted that some of these taxa need to be verified through additional data (Stadler and Hellwig 2005). Daranagama et al. (2016b) posed the question ‘Do xylariaceous macromycetales make up most of the Xylariomycetidae?. They compared macro- xylariaceous genera with micro-xylariaceous genera, i.e., those with inconspicuous ascomata and asexual anamorphs and provided a valuable table of the genera from both groups together with an extensive bibliography.

Since 2014 thirty new species of members of the Xylariaceae have been reported from Thailand alone: which is a reflection on the increased interest and research in these fungi especially in the tropics (Dai et al. 2014; Srihanant et al. 2015; Li et al. 2016; Tibpromma et al. 2017; Ju et al. 2018; Dayarathne et al. 2020; Konta et al. 2020; Wongkanoun et al. 2020; Wangsawat et al. 2021a).

An equally impressive number of species have also been added mainly from Southeast Asia and China by K.D. Hyde and co-workers (Dai et al. 2014; Li et al. 2016; Tibpromma et al. 2017; Dayarathne et al. 2020; Konta et al. 2020; Ma et al. 2023).

In Papua New Guinea the species composition of the Xylariaceae depended strongly on habitat characteristics. Overall, *Kretzschmaria*, *Nemania* and many *Xylaria* species occurred in dense, shaded sites, whereas the genus *Biscogniauxia* and several *Hypoxylon* species were more abundant in relatively dry and open sites. This is in general agreement with reports from Malaysia (Whalley and Whalley 2007), Thailand (Thienhirun 1997; Thienhirun and Whalley 2004), Mexico (Gonzalez and Rogers 1993). There are, however, problems in comparison of different regions because of the selection of different habitats within the ecosystem. In general, tropical Xylariaceae are good dispersers, and their preferred habitat characteristics differ largely between genera as well as species (Van der Gucht and Whalley 1996). We note that *C. tinctor* is the only member of the genus, which is widespread worldwide, and from our experience, it is the only species in which ascospores are readily germinated which might be part of the explanation. The importance of precise environmental habitat is well illustrated by *Engleromyces sinensis* and *R. diathrausta* (Rehm.) L.E. Petrini which both exhibit adaptations to life in cooler situations. They occur at high altitudes and the ascospores of *E. sinensis* can only be germinated at low temperature (Whalley et al. 2010) and those of *R. diathrausta* at temperatures below zero (Ouellette and Ward 1970). Stromata of *R. diathrausta* are regularly found on dry, weather exposed, on still-attached branches of alpine pines growing above 1800 m.asl (Petrini 2013).

### Chemical diversity and bioactivity

There is no question that the Xylariaceae provide a rich and diverse range of secondary metabolites. Many of these have been found to be novel and a considerable number have proved to exhibit bioactive properties (Stadler and Hellwig 2005). In their review (Helaly et al. 2018) noted the occurrence of 576 metabolites in the Xylariaceae and their related families and since then a range of other metabolites have been added by various authors as detailed by Becker and Stadler (2021).

Early studies on metabolites of the Xylariaceae reported that *Daldinia concentrica* produced perylene quinones (Allport and Bu’lock 1958, 1960) and *Rosellinia necatrix* cytochalasin E (Aldridge et al. 1972). These were followed by a series of publications by Edwards and his collaborators, at first extracting from stromata, then followed by broth cultures grown under static conditions for 6 weeks at room temperature. The compounds reported included the butyrolones (Edwards and Whalley 1979), serpenone (Anderson et al. 1982a), chestersiene (Anderson et al. 1982b), dihydroisocoumarins (Anderson et al. 1983), puntaporonines (Anderson et al. 1988), cubensic acid (Edwards et al. 1991). The presence or absence of these metabolites and others were shown to be of taxonomic importance as summarised by Whalley and Edwards (1995). Recognition of their significance in the classification of the Xylariaceae subsequently resulted in many significant publications on metabolites of the Xylariaceae based on high performance liquid chromatography coupled with diode array detection and mass spectrometric detection (HPLC–DAD/MS) in Stadler and Hellwig (2005) and Stadler and Fournier (2006). This has resulted in the discovery of over 150 secondary metabolites (Stadler and Hellwig 2005; Stadler 2011; Kuhnert et al. 2017; Helaly et al. 2018). The addition of chemical data to multigene sequences has now provided a comprehensive understanding of relationships within the Xylariaceae and their relatives and resolved several uncertain aspects in their existing classification (Wendt et al. 2018). Several bioactive metabolites have been reported from Xylariaceae and relatives that have been collected from Thailand such as mero-type triterpenoids, pyrazinoquinazolinone alkaloids and cytochalasin derivatives (from *X. humosa* Llyod, *X*. cf. *cubensis* (Mont.) Fr.PK108, *X*. cf. *cubensis* SWUF08-86, *X. allantoidea* (Berk.) Fr. SWUF76, and *X*. sp. SWUF08-37) all of which exhibited a degree of toxicity /against human cancer cell lines (KB, MCF-7 and NCI-H187) (McCloskey et al. 2017; Noppawan et al. 2020; Sawadsitang et al. 2015, 2018; Sodngam et al. 2014). Several isocoumarin and chromones from *X*. sp. SWUF09-62 showed both cytotoxicity against cancer cells and strong anti-inflammatory activities (Patjana et al. 2021). There is a hypothesis that chronic inflammation may lead to the initiation of cancer, meaning that a compound having both activities could be a chemoprevention and chemo-therapeutic drug candidate. Recent research published on former members of the Xylariaceae, belonging to the genera *Biscogniauxia* and *Annulohypoxylon*, reported several compounds including diorcinol, cordyol C, violaceol I, aspergillusene A from *Annulohypoxylon stygium* (Lév.) Y.-M. Ju, J.D. Rogers & H.M. Hsieh SWUF09-030 and bergamotene, guaiane, phthalide derivatives from *Biscogniauxia whalleyi* N.Wangsawat, C. Phosri and N. Suwanassai SWUF13-085 which showed both cytotoxic and anti-inflammatory activities (Pimjuk et al. 2021; Jantaharn et al. 2021). In addition, 2-hydroxyphenylacetic acid methyl ester, isolated from *Annulohypoxylon spougei* Suwannasai, M.P. Martin, Phosri & Whalley showed significant effects against both radish and ruzi grass radicle elongation, which were comparable to the commercial herbicide, glyphosate (Pimjuk et al. 2022). The Xylariales are a well proven source of bioactive compounds including antibiotics, antimalarials, antioxidants, anticancers, nemicidals, phytotoxins and for exhibiting other activities (Whalley 1996; Stadler and Hellwig 2005; Helaly et al. 2018; Wangsawat et al. 2021b). As stated by Helaly et al. (2018) the ‘Xylatiales contain genera which constitute one of the most prolific sources of secondary metabolites in the fungal kingdom’. They are arguably among the predominant fungal endophytes which are the producer organisms of pharmaceutical lead compounds including the antimycotic sordarins and the antiparasitic nodulisporic acids, as well as the marketed drug, emodepside (Helaly et al. 2018). It is, therefore, hardly surprising that with this impressive track record investigations of their metabolites are ongoing in many different parts of the world. Our studies on crude extracts from stromatic and endophytic *Xylaria* species in Thailand have indicated both promising antioxidant, antimicrobial and anticancer activities (Orachaipun et al. (2015a); Orachaipun et al. 2015b; Pharamat et al. 2013).

## Conclusions

The publication ‘The Xylariaceae: systematic, biological and evolutionary aspects (Rogers 1979)’ initiated an increased awareness about the Xylariaceae and Ascomycota in general. The monumental ‘Thoughts and musings on tropical Xylariaceae (Rogers 2000) stimulated a review on the xylariaceous mycobiota of Taiwan (Ju 2000) and has stimulated the publication of monographs of many genera, erection of new genera and descriptions of numerous new species. The recognition that their metabolites could be useful taxonomic markers (Whalley and Edwards 1995) led to extensive studies on their chemistry Stadler and Hellwig (2005) and their biological potential (Becker and Stadler 2021; Helaly et al. 2018). Chemical profiles in combination with multigene sequencing has resulted in a more natural and clearer classification of the family and related genera (Wendt et al. 2018). Importantly it has resulted in extensive surveys worldwide by resident mycologists, organization of specialist workshops and the training of numerous students. We now have a much greater understanding and appreciation of the Xylariaceae and relatives, Jack Rogers, we salute you.

## Data Availability

All information is included in this article.
